# Linear, Non-Conjugated Cyclic and Conjugated Cyclic Paraphenylene under Pressure

**DOI:** 10.3390/molecules24193496

**Published:** 2019-09-26

**Authors:** Miriam Peña-Álvarez, Samuele Fanetti, Naomi Falsini, Giulia Novelli, Juan Casado, Valentín G. Baonza, Mercedes Taravillo, Simon Parsons, Roberto Bini, Margherita Citroni

**Affiliations:** 1 School of Physics and Astronomy and Centre for Science at Extreme Conditions, University of Edinburgh, Edinburgh EH9 3FD, UK; 2 LENS–European Laboratory for Non-Linear Spectroscopy, 50019 Sesto, Florence, Italy; fanetti@lens.unifi.it (S.F.); nao89@hotmail.it (N.F.); roberto.bini@unifi.it (R.B.); margherita.citroni@nature.com (M.C.); 3 Centre for Science at Extreme Conditions and EastChem School of Chemistry, University of Edinburgh, Edinburgh EH9 3FJ, UK; G.Novelli@ed.ac.uk (G.N.); Simon.Parsons@ed.ac.uk (S.P.); 4 Department of Physical Chemistry, Faculty of Science, University of Málaga, CEI Andalucía Tech, Campus de Teatinos s/n, 29071 Málaga, Spain; casado@uma.es; 5 MALTA-Consolider Team, Department of Physical Chemistry I, Chemistry Faculty, University Complutense of Madrid, 28040 Madrid, Spain; vgbaonza@quim.ucm.es (V.G.B.); mtaravil@quim.ucm.es (M.T.); 6 Instituto de Geociencias IGEO (CSIC-UCM), 28040 Madrid, Spain

**Keywords:** linear and cyclic paraphenylene, pressure, vibrational spectroscopy, absorption, fluorescence

## Abstract

The *n*-paraphenylene family comprises chains of phenylene units linked together by C-C bonds that are between single- and double-bonded, and where *n* corresponds to the number of phenylene units. In this work, we compare the response of the optical properties of different phenylene arrangements. We study linear chains (LPP), cyclic systems (CPPs), and non-conjugated cyclic systems with two hydrogenated phenylenes (H_4_[n]CPP). Particularly, the systems of interest in this work are [6]LPP, [12]- and [6]CPP and H_4_[6]CPP. This work combines Raman and infrared spectroscopies with absorption and fluorescence (one- and two-photon excitations) measured as a function of pressure up to maximum of about 25 GPa. Unprecedented crystallographic pressure-dependent results are shown on H_4_[n]CPP, revealing intramolecular π-π interactions upon compression. These intramolecular interactions justify the H_4_[n]CPP singular optical properties with increasing fluorescence lifetime as a function of pressure.

## 1. Introduction

Since the discovery of conducting polyacetylene [1], organic molecules and polymers with a π-conjugated aromatic backbone have been the subject of much scientific attention due to their ability to transport charge and efficiently interact with light. The aim of the field of organic optoelectronics is to develop a broad range of deformable optoelectronic materials and devices. π-conjugated molecules undergo a wide variety of transformations under high pressure, such as the formation of aggregates and polymers [2,3], an area of chemistry that has been only lightly explored because of the difficulties of definitive in situ characterisation [4,5]. On the other hand, pressure can be used to modulate the optic-electronic properties of materials [6,7].

Benzene is one of the most versatile building blocks in optoelectronic materials as it can be arranged to form one- and two-dimensional nanomaterials, i.e., fullerene and graphene. In addition, at the molecular level, benzene can assemble into linear or cyclic configurations, forming polyaromatic systems. Notable examples are linear chains of aromatic units linked to each other by single C-C bonds as in linear paraphenylenes ([n]LPPs), where *n* is the number of phenyl units [8,9]. These materials have overtaken most of the field of optoelectronics materials and are used for applications in electronic and photo-electronic devices [10,11].

[n]LPPs in their ground state at ambient conditions have a non-planar configuration, presenting torsional angles between neighbouring phenyl units [12] and they become planar when a small pressure (<1 GPa) is applied [13,14,15,16]. Compression also leads to a decrease in the intermolecular distances, which has led Hanfland et al. [6] to suggest that above 5 GPa permanent intermolecular links could be formed from interactions between the π-systems of the 1D planar chains [17]. The increase of in-plane conjugation, which occurs on planarisation, and the compression of the intermolecular distances, results in a redshift in the luminescence spectrum of LPPs with pressure [6,13].

Cyclo[n]paraphenylenes ([n]CPPs) are formed by *n* phenyl units connected to each other through their para positions and forming a cyclic unit. These were first synthesised in 2008. The unusual cyclic molecular symmetry of [n]CPPs offers a unique case of cyclic circulation of the π-electron density, or cyclic π-conjugation [13,18,19], which confers on them distinct optical and electronic properties governed by their increasing strain with decreasing *n* [20,21]. The versatility of these systems in terms of aromaticity, unique size-dependent supramolecular properties and encapsulation of guest molecules leads to promising applications in materials science [22,23,24,25,26,27].

Due to the decrease in bending angle of the phenyl units with increasing size of the [n]CPPs, large [n]CPPs have features close to those of long [n]LPPs. However, large [n]CPPs are more energetic systems than long [n]LPPs on account of the strain generated in the cyclic systems by the deformation of planar phenyl units [28,29,30,31]. While in [n]LPPs the energy gap decreases with *n* [32,33,34], the HOMO-LUMO gap of [n]CPPs increases. However, their optical properties are dominated by the forbidden HOMO-LUMO transition [30,31,33,35,36,37].

Many different aspects of the ambient pressure chemistry of [n]CPPs have been already addressed in different laboratories [36]. In previous work, we explored high-pressure structural and mechanical properties of [n]CPPs [38,39,40]. The two smallest and most highly strained members of the series, [5]CPP and [6]CPP, polymerised at pressures between 5 and 6 GPa, whereas larger [n]CPPs are softer and remain in a monomeric state up to 10 GPa. [n]CPPs undergo ovalisation with compression at lower pressures with increasing sizes [40]. However, it is still unknown how the optical properties of [n]CPPs respond to deformation of the cycle.

**Figure 1:**
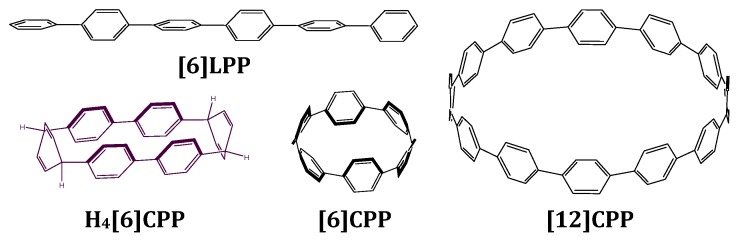
Scheme of the structures discussed in this work. [6]LPP stands for hexa linear paraphenylene, which is formed by six phenyl units linearly bonded to each other in their para position. H_4_[6]CPP stands for tetrahydro hexa cycloparaphenylene and is configured by two diphenyl units linked to each other through two cyclohexadienes. [6]CPP stands for hexa-cycloparaphenylene and [12]CPP stands for dodeca-cycloparaphenylene. These are formed by *n* phenyl units linked to each other in their para positions.

Optical studies of molecular systems under high pressure allow us to correlate how intermolecular interactions and structural changes affect photo-physical properties without modifying their chemical composition [41,42]. In this work, we describe a systematic study on the electronic properties of [n]CPPs, providing a thorough link between [n]CPPs and [n]LPPs. We attempt to explore the mechanical response of [12]CPP, [6]CPP with a cyclic configuration, [6]LPP with linear conjugation and the tetrahydro [6]CPP (H_4_[6]CPP) with a non-conjugated cyclic configuration (see Figure 1). We present the first crystallographic study of a non-conjugated cycle H_4_[6]CPP as a function of pressure. Infrared and Raman spectroscopies are used to evaluate the reversibility or irreversibility of the pressure-induced structural changes in the conjugation backbone. UV-vis absorption and one- and two-photon excitation spectroscopies, also at high pressure, are used to reveal the striking optical properties of the different phenyl configurations. Pressure-dependent fluorescence lifetimes were also measured in some cases. As the evolution of [n]LPPs structural and optoelectronic properties with pressure is well understood [6,13,16], [6]LPP will be used as a reference and to validate our measurements.

## 2. Results

### 2.1. Effect of Pressure on the Crystal Structure

In phase II benzene, the aromatic rings are arranged in a slipped-parallel configuration, i.e., the molecules lie on parallel planes [43]. The compression of benzene phase II induces a decrease in the intermolecular distances, resulting in covalent intermolecular bonds shorter than 3.0 Å at around 30 GPa [43,44]. Through X-ray diffraction (XRD) experiments, Heimel et al. [17] found that LPPs’ compression decreases intermolecular distances from about 4.045 Å [45] to about 3.5 Å at 6 GPa (see Table S1). Therefore, it is expected that the compression of [6]LPP would lead to the formation of intermolecular covalent linkages.

[12]CPP, whose diameter is about 1.65 nm, has a crystal structure characterised by a T-shaped arrangement of the molecules, which strongly impedes π-π intermolecular contact formation (see Table S2). [6]CPP, with a diameter of about 0.88 nm, is polymorphic and either forms tubular or herringbone arrangements [46]. Pressure-dependent XRD studies of these systems are beyond the scope of this work.

**Figure 2:**
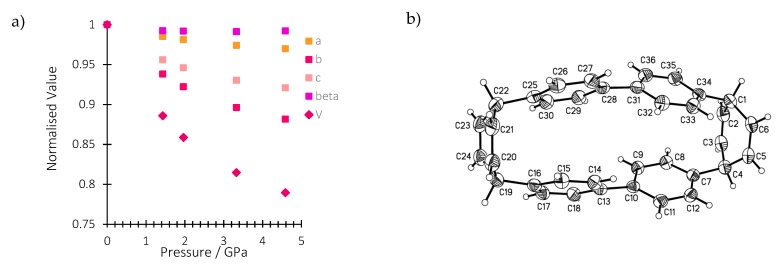
(**a**) Variation of the normalised lattice parameters and unit cell volume of H_4_[6]CPP as a function of pressure. (**b**): H_4_[6]CCP structure in the crystal at ambient pressure and 298 K. Ellipsoids are shown with 30% probability surfaces.

In this work, we conduct a pressure-dependent X-ray diffraction study on a single crystal of H_4_[6]CPP in order to relate structural changes with the spectroscopic changes observed upon compression. Table S3 reports all the experimental details. The crystal structure of H_4_[6]CPP was investigated using single-crystal X-ray diffraction between ambient pressure and 4.59 GPa. H_4_[6]CPP crystallises in the monoclinic space group *P*2_1_/*c*, with four molecules per unit cell and one molecule in the asymmetric unit. The effect of pressure on the unit cell parameters is shown in Figure 2a. The cell axes and volume smoothly decrease with each step in the pressure series, showing the absence of any first-order phase transitions. The *a*, *b* and *c*-axes compress by 3.1%, 13.4% and 8.6%, respectively, up to 4.59 GPa.

The molecular structure is shown in Figure 2b. The closest intramolecular distance between phenyl units under ambient conditions is formed between the phenyl rings containing C13 and C25: the centroid–centroid distance is 3.79 Å and the angle between the planes is 10.59(9)°. At 4.59 GPa, these geometries change to 3.33 Å and 6.1(7)°, respectively. In the same pressure range, the length of the molecule as measured by the distance between the centroid of the hexadiene rings based on C1 and C19, undergoes a modest change from 10.65 to 10.69 Å such that the molecule becomes more oblate overall.

**Figure 3:**
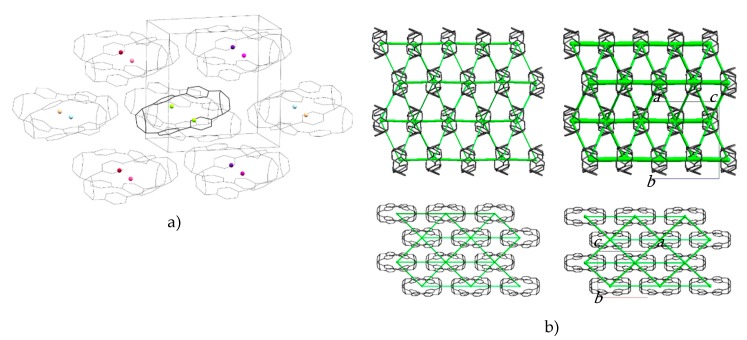
(**a**) Crystal packing in H_4_[6]CPP. The molecular centroids are shown as small spheres where the colours correspond to the molecular contact energies listed in Table 1. (**b**) Dispersion energy framework of the H_4_[6]CPP structure at 0 GPa (left) and 4.59 GPa (right) viewed along the *a*-axis (top) and *c*-axis (bottom). The thickness of the struts is proportional to the magnitude of the energy. Hydrogen atoms are removed for clarity.

The molecules are packed in a distorted body-centred cubic topology in which a central reference molecule is surrounded in a plane by six others, with four additional molecules in the planes above and below, giving an overall molecular coordination number of 14 (Figure 3a). This arrangement persists throughout the pressure range studied (Table S4 lists extended structural information). An analysis of the intermolecular energies is shown in Table 1, where colours shown for each contact are also indicated in the molecular centroids of Figure 3a.

**Figure 4:**
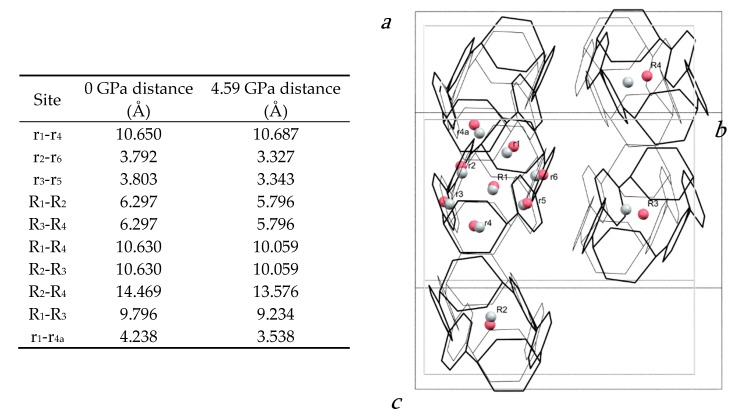
(**left**) Table of centroid–centroid distances in the H_4_[6]CPP structure at 0 and 4.59 GPa. (**right**) Structure of H_4_[6]CPP viewed along a* at ambient pressure (black wireframes) and at 4.59 GPa (grey wireframes), indicating intramolecular and intermolecular distances calculated between centroids (pink for 0 GPa and grey for 4.59 GPa). Hydrogen atoms are removed for simplification. Molecular centroids are indicated with R_1_, R_2_; the centroids of individual rings are labelled r_1_, r_2_.

The pattern of intermolecular contacts is shown using energy frameworks in Figure 3b. The lack of framework struts in the *b*-direction is presumably the reason why the crystal experiences most compression along this direction (Figure 2a). The strongest interaction is a non-specific dispersion contact formed by alignment of the long axes of two molecules; thus, at −50 kJ mol^−1^, the energy of this interaction is like a moderately strong hydrogen bond. The weakest interactions (the pale blue contact with −10.7 kJ mol^−1^ and the yellow contact with −4.8 kJ mol−^1^) are built along the *a*-axis, where the facing phenylene ring fragments from different molecules interact with each other through their respective double bonds. These interactions are also the shortest, with the ring centroids being separated by 4.24 Å at ambient pressure and 3.54 Å at 4.59 GPa. The variation of other intra- and intermolecular ring and centroid distances are shown in Figure 4. The fact that at 4.59 GPa the shortest intramolecular distance is 3.33 Å while the shortest intermolecular distance is still 3.54 Å suggests that further compression could lead to the formation of covalent intramolecular links.

### 2.2. FTIR

**Figure 5:**
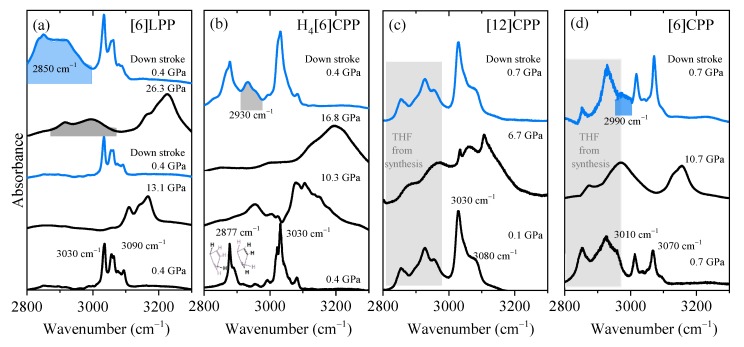
Infrared spectra at selected pressures of the different crystalline compounds measured using KBr as the pressure transmitting media. Blue spectra correspond to the product obtained in decompression and the product band is highlighted: (**a**) [6]LPP; (**b**) H_4_[6]CPP; (**c**) [12]CPP; and (**d**) [6]CPP.

It has been demonstrated that the most significant spectral changes occurring in paraphenylenes as a function of pressure are seen in the C-H stretching modes as they are sensitive to the sp^3^ or sp^2^ hybridisation of carbon [47,48]. The clearest example is provided by the pressure-induced polymerisation of benzene itself in which the C-H stretching from aromatic benzene at around 3000–3200 cm^−1^ in the IR spectrum vanishes in decompression to 27 GPa and is replaced by a C-H stretching band characteristic of saturated C(sp^3^) at around 2900 cm^−1^ [47,48]. Therefore, this section will mainly discuss this high-frequency region, although data have been collected in the frequency range between 500 and 3200 cm^−1^ and are available in the Supporting Information (Figures S1–S4). In Figure 5, the FTIR spectra in the 2800–3300 cm^−1^ region at selected pressures are shown in order of increasing cyclic conjugation: [6]LPP, H_4_[6]CPP, [12]CPP and [6]CPP.

FTIR experiments were performed to explore the possibility of pressure-induced reactivity. In addition, IR experiments were used to calibrate the frequencies of the vibrational modes as a function of pressure and to use them as an internal pressure gauge in the fluorescence measurements. For each of the phenylene systems, one or two polycrystalline samples were investigated using ruby fluorescence as a pressure marker [49].

In Figure 5a, we show the FTIR spectra at different pressures for [6]LPP in the region corresponding to the C-H stretching modes. With increasing pressure, there is a change in the C-H profile that can be related to the change from a twisted to a planar configuration (see Figure S1). When the maximum pressure reached is below 15 GPa, decompression re-establishes the initial [6]LPP D_2_ configuration. However, if [6]LPP is compressed above 25 GPa, then a saturated C-H stretching mode at around 2900 cm^−1^ appears in the infrared spectrum. This new band rapidly grows to become the most intense feature of the spectrum after the pressure is released. These results indicate that compression at around 25 GPa can induce the loss of aromaticity within the phenyl units through the formation of intermolecular linkages.

Figure 5b shows the pressure-dependent FTIR of H_4_[6]CPP. At the lowest pressure, the H_4_[6]CPP high-frequency spectrum is characterised by two sets of bands: one at lower frequencies (around 2887 cm^−1^ and 2900 cm^−1^) that correspond to C-H stretching modes of saturated carbons and of those from the olefinic C-H bonds of the cyclohexadiene units, respectively. Bands at 3030 cm^−1^ correspond to the C-H stretches of the phenylene units. As seen in Figure 5b, compression of H_4_[6]CPP leads to an intensity increase of the C-H stretching band derived from the phenylene moieties, which could be interpreted by the pressure-induced strengthening of the π-π interactions as we know that the intramolecular distances are decreasing in compression. Interestingly, decompression from 17 GPa shows the irreversibility of the process as there is a new contribution around 2930 cm^−1^ that is consistent with an increase in C-H stretching involving C with higher sp^3^ character. Such reactivity might be induced by intra- or intermolecular reactivity through the phenylene units, or through the cyclohexadiene units, respectively. The shorter intramolecular distances of 3.33 Å at 4.59 GPa and obtained in the XRD results indicate that intramolecular reactivity might be favoured in this case.

It has been reported that compression of [12]CPP induces the deformation of the cycle towards a more oval configuration, creating shorter and longer diameters [7,40]. The onset of ovalisation is below 1 GPa [40]; however, the FTIR spectra presented in Figure 5c show that compression within the oval regime does not seem to affect the sp^2^ framework as the C-H stretching modes broaden and upshift while maintaining their band shapes. After pressure is released, the spectrum is identical to that of the pristine sample.

The compression of tubular [6]CPP above 6 GPa causes a permanent deformation of the cycle that could lead to the formation of intermolecular covalent linkages [39]. Figure 5d shows that at 10 GPa, the C-H stretching modes of the aromatic phenyl units upshift and broaden but their overall profile persists. After decompression, a new contribution at lower frequencies appears at around 2990 cm^−1^, indicating the irreversibility of the compression cycle. This band would correspond to a contribution from more saturated carbons and could be explained by a pressure-induced polymerisation.

The pressure shift of aromatic C-H stretching has been estimated for all the systems and compared with that of benzene in Table 2. As seen in the table, benzene and [6]LPP show similar values, which in increasing order are as follows: benzene, [6]LPP, [6]CPP, H_4_[6]CPP and [12]CPP. It is interesting that [12]CPP is the one with largest coefficient as it is the only one showing a reversible response to compression within the explored pressure range.

### 2.3. Raman Spectroscopy

**Figure 6:**
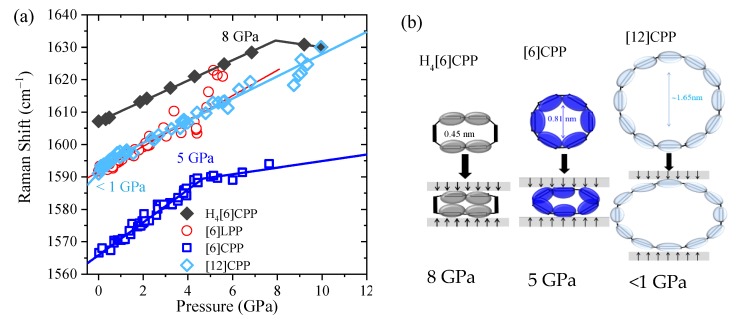
(**a**) Pressure dependence of the Raman shifts GA_1g_ mode of the different crystalline compounds with no pressure transmitting media. Solid lines correspond to the data fitting to Equation (1). (**b**) Scheme representing deformation of the cycles at *P_oval_*.

Pressure-induced intramolecular interactions can be responsible for the general increase in the positions of the Raman bands. It has already been established that there is a common Raman response that reveals structural ovalisation of [n]CPP. The deformation of the cycle is revealed by a change of the rate of frequency shift as a function of pressure [40]. The most intense Raman bands appear in the 1560–1600 cm^−1^ range, having originated from symmetric vibrations arising from collective C-C stretching modes of the benzenoid rings along the transversal direction. Different sp^2^ carbon systems as single-wall carbon nanotubes or graphite also present C-C stretching modes in the 1560–1600 cm^−1^ region; therefore, we will refer to them as ‘G_A1g_’ modes (G is aligned to graphite, which also shows this mode, and A_1g_ to the symmetry) [7]. In this work, we measure the Raman spectra of selected powdery systems within an anvil cell at selected pressures. The Raman shift GA_1g_ [7] is shown for all of the systems in Figure 6 (see also Figures S5–S8 for raw data and analysis of the decompressed sample). As in the compression of phase II of benzene [45], and in line with former results, the GA_1g_ mode of [6]LPP linearly upshifts [16,50]. The pressure at which the [n]CPPs change the slope and, thus, the cycle, becomes oval is higher the more strained and rigid the cycle is, in agreement with the change in slope at around 5 GPa in [6]CPP and below 1 GPa in [12]CPP [40]. In the case of the non-conjugated cycloparaphenylene H_4_[6]CPP, the C-C stretching mode from the aromatic units of H_4_[6]CPP linearly upshift to approximately 8 GPa, where there is a change in slope. Figure 6b shows the described deformations for the conjugated and non-conjugated CPP.

Equation (1) describes approximate piecewise linear trends in the Raman frequencies:

(1) $$\begin{array}{ll} \omega_{i}(P)=\omega_{i}(0)+A_{,i}P, & \mathrm{if}\;(P<P_{oval}^{n}),\\ \omega_{i}(P)=\omega_{i}(0)+A_{,i}P_{oval}^{n}+B_{,i}(P-P_{oval}^{n}), & \mathrm{if}\;(P>P_{oval}^{n}) \end{array}$$

A_,i_ and B_,i_ correspond to the pressure coefficients at pressures below and above $P_{oval}^{n}$ (ovalisation pressure), respectively, and ω_i_(0) is the Raman shift at ambient pressure. Thus, for each band we have three parameters, (ω_i_(0), A_,i_ and B_,i_) and these are compared in Table 3. The similar of A_.i_ for benzene [45], [6]LPP and H_4_[6]CPP demonstrate their similar physical, chemical, and structural response to compression. In previous work, we demonstrated that the similar large values of the *A* coefficients for CPPs are related to the deformation of the cycle and, therefore, of the phenyl units [40]. Moreover, the H_4_[6]CPP C=C stretching mode corresponding to the cyclohexadiene units linearly upshift to 10 GPa while not showing a change in slope.

### 2.4. Absorption and Fluorescence at Low Pressures

**Figure 7:**
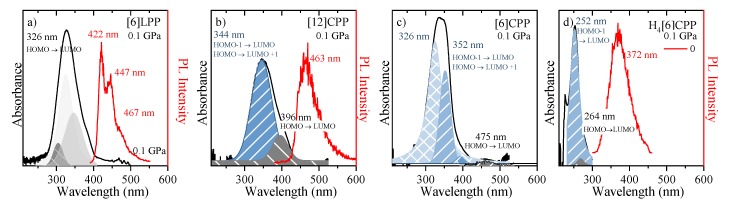
UV-vis absorption and emission spectra are marked as black and red, respectively. (**a**) [6]LPP; (**b**) [12]CPP; (**c**) [6]CPP; and (**d**) H_4_[6]CPP. All samples were measured as powdered. HOMO-LUMO transitions are marked. [6]CPP emission is not allowed by selection rules being this why there is not PL intensity indicated (see text).

In linear paraphenylenes, the absorption spectrum is a consequence of the allowed HOMO-LUMO absorption. The HOMO-LUMO band gap in [n]LPPs decreases with the lengthening of the conjugation that occurs as *n* increases, but it can also be enhanced by the decrease of inter-phenyl torsional angles [53,54]. Thus, the absorption spectrum of [6]LPP can be fitted to three different vibronic contributions, the 0-0, 0-1 and 0-2 as shown in Figure 7a. [6]LPP is a good light emitter, with a lifetime value of 0.78 ns [55]. The PL spectrum of [6]LPP is characterised by a vibronic progression with three main contributions at 422, 447 and 467 nm. The highest energy band at 422 nm is the 0-0 transition, followed by 0-1 and 0-2.

In the case of the [n]CPPs, the complex interplay of symmetry, π-conjugation, conformational distortion and bending strain controls the photophysical properties. These molecules have high optical absorbance in the blue spectral region that increases with the ring size; however, the absorption maxima (at ~340 nm) are independent of the ring size as seen in the absorption spectra of [12]- and [6]CPP shown in Figure 7b,c, respectively. Vertical absorption happens between the HOMO-1/HOMO-2 to LUMO, HOMO to LUMO+1/LUMO+2 and S_2_ and S_3_ states, respectively, which are degenerate (or almost-degenerate in [6]CPP) by symmetry [22].

The absorption spectra of both CPPs contain shoulder-like bands at around 396 nm in [12]CPP and 475 nm in [6]CPP, which correspond to the HOMO-LUMO absorption S_0_ to S_1_ [56]. By contrast to the LLPs, a higher value of *n* implies a larger HOMO-LUMO band gap [30,31]. Although these HOMO-LUMO transitions are forbidden by symmetry, the imperfect geometry of CPPs leads to small perturbations in the electronic wave function that result in a small but non-zero oscillator strength and the presence of HOMO-LUMO absorption at low intensity [55]. The inhomogeneous broadening of the [6]LPP and [n]CPP spectra is due to conformational structural variations related to irregular variations of the dihedral angles [30,31,55].

A peculiar feature of cycloparaphenylenes is their unusual optoelectronic behaviour as a function of molecular size. Specifically, the emission and quantum efficiency diminishes, which lengthens fluorescence lifetimes for smaller *n* [35]. This diminution in quantum efficiency implies that the [6]CPP S_1_ state is not fluorescent [57]. We find that the emission of [12]CPP shows a maximum at 463 nm and lifetime of 2.6 ns, which is in agreement with previous results [35]. The explanation for the observation of this emission from the forbidden S_1_ state has been approached through a variety of hypotheses: phonon-assisted transitions [36]; efficient fluorescence in large CPP hoops to a broken Condon approximation due to exciton self-trapping [55]; vibrational intensity borrowing from the higher states [58]; Jahn−Teller distortion effects due to coupling to circle-to-oval vibrational modes breaking the selection rules [37]; and strong exciton−vibration couplings [36,55]. None of these occur in the small molecules, which remain inefficient emitters [55].

While the S_0_→S_1_ transition is forbidden in the linear response, it can be probed by nonlinear spectroscopies such as two-photon absorption [36]. In this work, one-photon (OP) and two-photon (TP) absorption were tested for both [12]- and [6]CPP. Emission is a one-photon transition and its activity is not affected by the excitation mechanism (OP or TP). Thus, in the case of [12]CPP, no significant change in the PL spectrum is observed, which is in agreement with previous results [30,36]. However, in the [6]CPP case, no fluorescence was detected.

The absorption and emission spectra of H_4_[6]CPP are presented in Figure 7d. The absorption UV-vis spectrum was measured in a KBr matrix. The band profile analysis of the absorption spectrum shows that it is formed from two contributions. To understand the origin of these two contributions, we conducted TD-DFT B3LYP/6-31G(d,p) calculations on the optimised B3LYP/6-31G(d,p) structure of a single molecule. These calculations show that there is only one absorption allowed that would correspond to the maximum of the absorption band at 252 nm corresponds mainly to the HOMO-1 to LUMO/HOMO to LUMO+1 transition with an oscillator strength of 0.91. On the other hand, the HOMO-LUMO absorption is formally symmetry-forbidden absorption (see supporting information for more information); however, we relate the experimentally observed band at 264 nm to this HOMO-LUMO transition. As in the case of [12]CPP, in symmetry distortion of H_4_[6]CPP should be responsible for perturbing symmetry rules, which explains why the HOMO-LUMO absorption is observed. On the other hand, the fluorescence spectrum at low pressure was measured through one-photon excitation within the diamond anvil cell at 0 GPa. As seen in Figure 7d, H_4_[6]CPP is a strong fluorophore whose lifetime is 10 ns, which is lower than that found for diphenyl units at 16.0 ns [55] as expected for systems with larger π-conjugation.

### 2.5. Optical Absorption in Compression

The UV-visible absorption spectra in the region of 370–500 nm were measured for all the samples at selected pressures. Powdered samples were diluted with NaBr and loaded into the gasket chamber together with a ruby chip, whose fluorescence shift was used as pressure marker [48]. As reference in the absorption measurements, the sample chamber was loaded with NaBr and its UV-vis transmission spectrum was measured.

**Figure 8:**
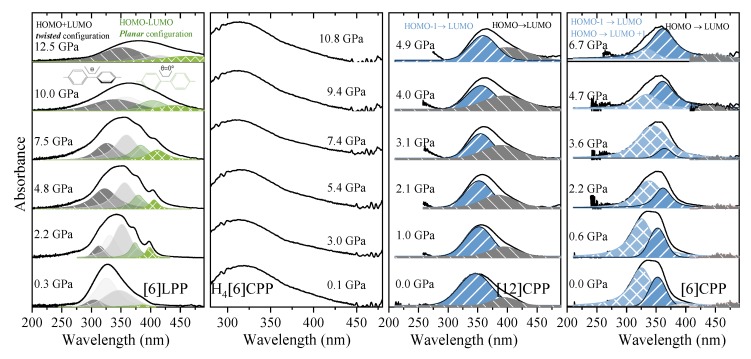
Absorption spectra of powdered samples in the near-UV-vis spectrum at selected pressures (diamond absorption has been subtracted). (**a**) [6]LPP: different contributions have been fitted to Voigt profiles for the vibronic pattern. Grey contributions correspond to the configuration with twisted neighbouring phenylenes and green to the planar configuration. (**b**) Absorption edge for H_4_[6]CPP using H-silicon carbide as anvils. (**c**) Absorption of spectra of [12]CPP. Grey contribution corresponds to the forbidden transition to the S_1_ state produced through the HOMO-LUMO transition and blue to the transitions to the S_2_ and S_3_ states corresponding to the HOMO-1 -LUMO, HOMO-LUMO+1 configuration change. (**d**) Absorption spectra of [6]CPP. Blue contributions correspond to non-degenerate transitions to S_3_-S_4_ and to S_2_, which at 0 GPa are at around 320 and 360 nm, respectively. Grey contribution corresponds to the forbidden transition to the S_1_ state HOMO-LUMO transition.

UV-vis measurements of [6]LPP up to 12.5 GPa (Figure 8a) show a broadening of the absorption bands with pressure, accompanied by new contributions that appear at lower energy (marked in green). Computational work demonstrates that the band gap decreases with the decreasing angle between neighbouring phenyl units [49]. With support from TD-DFT calculations (Figure S10), additional absorption bands at lower energy can thus be assigned to the planar conformer. The absorption intensity rapidly decreases with compression although spectra could not be measured beyond 12.5 GPa as seen in Figure 8a.

The H_4_[6]CPP pressure-dependent absorption measurements were done using H-silicon carbide as anvils, which limits the spectral range to 300 nm and above, and only part of the S_0_→S_1_ absorption 252 nm could be detected. In Figure 8b, the absorption edges at different pressures are presented.

Figure 8c shows the absorption spectra of [12]CPP at selected pressures. In addition to the redshift of the bands, the contribution from the S_0_→S_1_ transition increases in intensity on compression. This agrees with the already described pressure-induced ovalisation of the cycle, which would perturb the symmetry of the excited S_1_ state. Indeed, in the case of [6]CPP, whose pressure-induced deformation occurs at around 5 GPa[39], in the visible region at around 4.7 GPa, the S_0_→S_1_ contribution seems to grow in intensity (Figure 8d). However, the S_0_→S_1_ intensity above *P_oval_* is not as high as expected for the completely allowed transition. Moreover, pressure seems to change the relative intensities of the bands for the S_0_→S_2_ and S_0_→S_3_ transitions. While in [12]CPP pressure favours π-conjugation [40], in [6]CPP pressure leads to a lowering of symmetry within the cycle leading to its polymerisation [39,40].

**Figure 9:**
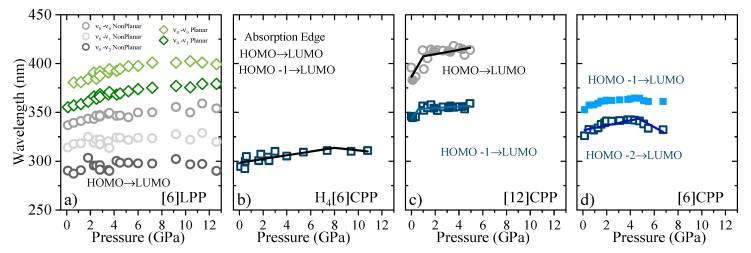
Pressure evolution of the absorption maxima for different crystalline compounds. (**a**) [6]LPP: the different vibronic transitions are reported. Grey contributions are assigned to the configuration with twisted neighbouring phenylenes and green to the planar configuration. (**b**) Absorption edge for H_4_[6]CPP. (**c**) Absorption of [12]CPP. Grey contribution corresponds to the forbidden S_0_→S_1_ transition, HOMO-LUMO, and blue to the S_0_→S_2_ and S_0_→S_3_, HOMO-1 -LUMO and HOMO-LUMO+1, respectively. (**d**) Absorption of [6]CPP, where blue contributions correspond to the non-degenerate S_0_→S_3_-S_4_, and S_0_→S_2_. For [6]CPP, the S_0_→S_1_ transition could not be followed as a function of pressure due to its negligible intensity.

In Figure 9, the shift of the absorption bands as a function of pressure is shown for the four systems discussed here. For all the phenylene systems, the absorption redshifts with pressure as a result of a decrease in the band gap. However, the rate at which this occurs is strongly dependent on the π configuration. Table 4 reports the coefficients of the absorption shift estimated for all the systems studied here. In systems with large π conjugation, such as picene, the S_1_ band has been observed with a redshift of ~8 nm/GPa. This energy gap closure is a result of the decrease in the intramolecular distances involving π-π interactions [59]. The torsional freedom of [6]LPP compared with that of picene leads to subtle changes in which the absorption bands redshift by ~1 nm/GPa. In the case of H_4_[6]CPP, there are slight changes in the slope of the absorption edge going from ~2 nm/GPa to ~−1 nm/GPa at around 8 GPa, which can be associated with the pressure-induced interactions between intramolecular biphenyl as inferred from the FTIR and Raman observations. As such, [6]LPP and H_4_[6]CPP present analogous trends, whereas the pressure-induced interactions between cofacial phenyl units in the cycle deviates the slope (see FTIR section).

Interestingly, in the case of [12]CPP, both the S_0_→S_2_ and S_0_→S_1_ transitions redshift with compression, presenting a change in the slope at around 1 GPa, *P_oval_*, going from ~15 nm/GPa and ~30 nm/GPa to ~1 nm/GPa and ~2 nm/GPa, respectively. These large slopes indicate important configurational rearrangements induced by pressure, as previous theoretical calculations estimated a band decrease of 10 nm/GPa in the order of magnitude of the results we obtain [39,40].

In the case of [6]CPP, the S_0_→S_2_ and the S_0_→S_3_-S_4_ transitions at around 5 GPa show a variation in the trend from red-shifting to blue-shifting: the S_0_→S_2_ goes from ~4 nm/GPa to ~−5 nm/GPa and the S_0_→S_3_-S_4_ from ~8 nm/GPa to ~−9 nm/GPa. At pressures above *P_ova_l*, [6]CPP is expected to become highly strained and deformed, with shorter intermolecular interactions [39], which explains the sudden change in the absorption trends.

### 2.6. Fluorescence in Compression

**Figure 10:**
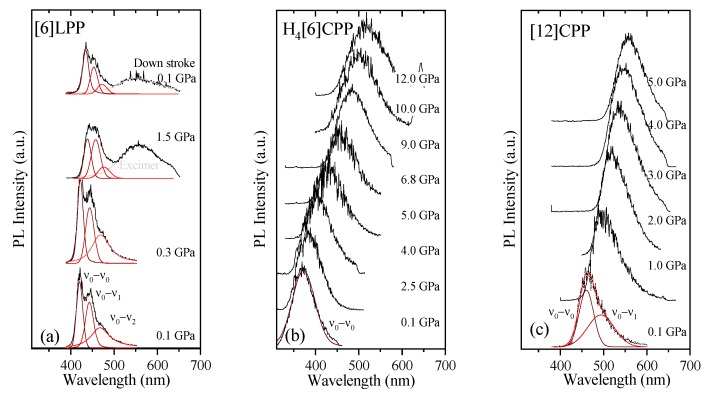
Fluorescence spectra at various pressures at 300 K for the different crystalline compounds: (**a**) [6]LPP; (**b**) H_4_[6]CPP; and (**c**) [12]CPP. Spectra were fitted using Voigt profiles.

**Figure 11:**
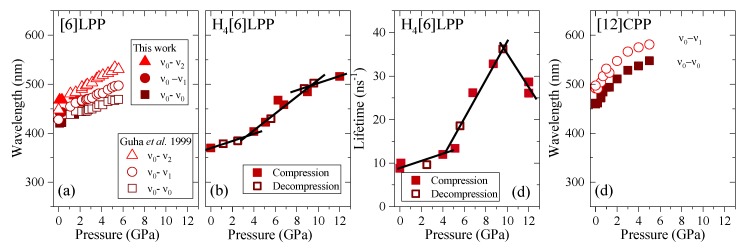
Emission data represented as a function of pressure for the different crystalline compounds. (**a**) Transition energies of [6]LPP: empty symbols correspond to the experimental results from Guha et al. [60]. (**b**) Transition energy (0-0) of H_4_[6]CPP. (**c**) Fluorescence lifetime of H_4_[6]CPP. (**d**) Transition energies of [12]CPP.

Fluorescence measurements were performed on polycrystalline samples loaded with different PTM. To avoid sample irradiation, pressure was measured through the calibrated FTIR shifts measured in the previous section; thus, the cell had to be moved between pressure points to the different setups. The dependence of the fluorescence signal intensity upon the laser power is linear or quadratic depending on whether the fluorescence follows a one-photon (OP) or two-photon (TP) absorption process. Thus, at each pressure step, the fluorescence intensity dependence was measured to determine the process type. The fluorescence spectra and pressure-trends for the three systems are shown in Figure 10 and Figure 11. Table 5 gathers the pressure coefficients of the bands estimated for all the systems studied here.

TP fluorescence spectra of [6]LPP were measured using 740 nm as an excitation line. The fluorescence spectra of [6]LPP are characterised by the S_1_→S_0_ emission, which shows a well-defined vibronic pattern. [6]LPP initially was loaded without a PTM; however, during the high-pressure fluorescence experiments of [6]LPP, the formation of an excimer at pressures below 2 GPa was characterised by a fluorescence band at around 550 nm. The excimer formation is a very interesting result because it has strong intensity at very low pressure. This behaviour is different to benzene, which showed a sudden intensity exchange between the monomer and excimer transitions at around 6.1 GPa [61]. Here, formation of the excimer is likely due to the presence of defects among the crystal grains and not the reactivity trigger as IR showed much higher pressures are required. Consequently, it was necessary to work with an inert PTM to avoid the formation of such defects. In these conditions, the excimer was still present. On the other hand, as seen in Figure 10a, the different vibronic transitions are observed as they all redshift with increasing pressure. Figure 11a shows the shift of the three vibronic contributions as a function of pressure. Our results are in excellent agreement with those of Guha et al. [59]. All the data reported show a significant band gap decrease with increasing pressure, with an average linear pressure coefficient 2 nm/GPa calculated above planarisation (ca. 1 GPa), which is in agreement with the absorption measurements.

In H_4_[6]CPP, fluorescence spectra were measured using different excitation wavelengths between 540 and 660 nm. The spectra at selected pressures are presented in Figure 10b. In contrast to [6]LPP, H_4_[6]LPP fluorescence is a broad single band, which is assigned as a 0-0 vibronic transition. The TP excitation profile was also measured at selected pressures, detecting fluorescence around its maximum. As in the absorbance experiments, H_4_[6]CPP shows a significant redshift in compression at about 150 nm in the 12 GPa range (Figure 11b). Moreover, the lifetime of the S_1_→S_0_ emission was measured as a function of pressure and a striking increase in lifetime is observed, going from 9 ns at low pressure to a maximum of 38 ns at 9 GPa as shown in Figure 11c. Measurements in compression and decompression were conducted to ensure reversibility of the process. Interestingly, unlike in [6]LPP, three regimes are observed: 0–4 GPa with a coefficient of ~5 nm/GPa and lifetime increase of ~0.8 ns/GPa; 4–9 GPa with a redshift coefficient of ~20 nm/GPa and lifetime increase of ~5 ns/GPa; and a third regime up to 12 GPa in which the coefficient is smaller with ~8 nm/GPa and lifetime decrease from a rate of −3 ns/GPa. This change in rate coincides approximately with the maximum pressure for which X-ray data could be collected. The diffraction data at 4.59 GPa are much weaker than at lower pressures, which may point to gradual transformations. These results in the change in the optical properties with pressure confirm that H_4_[6]CPP undergoes intramolecular changes involving π-π interactions. This can be interpreted as the increase in the dipole moment of the excited state with stronger π-π intramolecular interactions causing a decrease in the transition energies [62]. At pressures above 9 GPa, further compression leads to stronger intramolecular connections that do not seem to be so energetically favoured as the energy gap does not decrease so steeply, whereas compression up to 12 GPa seems to lead to a product without covalent bond formation as the process is fully reversible.

In Figure 10c, the TP fluorescence spectra of [12]CPP at selected pressures are presented. These were measured with an excitation line of 760 nm. The asymmetry in the fluorescence profile indicates that the spectra are formed by at least two different contributions as previously described [35,63]. We have fitted the spectrum to two contributions where the energy spacing between these peaks is approximately 40 nm, corresponding to approximately 1450 cm^−1^ and matching the phenylene stretching vibration [64]. [12]CPP fluorescence shows a redshift of the same order of magnitude as in the absorption measurements (Figure 11d). The average shift of the vibronic modes gives a redshift of 50 nm/GPa up to 1 GPa and of 10 nm/GPa above 1 GPa. These results are in line with the compression of a low-pressure rigid phase in which pressure would favour the cyclic conjugation [40,49,65].

[6]CPP fluorescence was measured with OP and TP excitations, and pressures below and above ovalisation pressure. However, significant fluorescence could not be detected for any configuration. This lack of results indicates that the deformation of the cycle does not allow the S_0_→S_1_ transition and confirms the fact that the absorbance observed in the bigger cycles at room pressure cannot be related with a Jahn–Teller effect [37].

## 3. Discussion

In this section, the results obtained through different diagnostics are presented together. The discussion will be presented individually for each of the systems by describing the main spectral changes observed during compression and how these are interpreted. Table 6 summarises the main points.

[6]LPPs. Around the 1–2 GPa range, these conduct planarisation towards their D_2h_ symmetry. At the same time, compression induces continuous reduction of the lattice parameters through the intermolecular distance decrease [16,17]. Absorption and fluorescence data of up to 12 and 6 GPa, respectively, indicate that pressure induces a band gap reduction. However, this reduction is not as large as expected if π-π interactions were strongly favoured. This is related to the fact that [6]LPP molecules are arranged in a herringbone structure within which the neighbouring molecules are not coplanar and, consequently, higher pressures than 12 GPa are required to facilitate their interactions. Fourier Transformation Infrared (FTIR) spectroscopy demonstrates that pressures of 23 GPa lead to the formation of a system characterised by an intense C-H stretching band typical of saturated systems. Consequently, we can consider that pressure induces shortening between intermolecular distances that at around 23 GPa leads to the formation of intermolecular linkages, generating a 3D π-bonded structure.

H_4_[6]CPP. Our XRD experiments demonstrate that compression leads to an important decrease within the intramolecular distances up to 4.59 GPa. Therefore, it could be expected that further compression would facilitate the formation of intramolecular interactions by shortening the biphenyl intramolecular distances. In this sense, compressions at 12 and 10 GPa are shown to be fully reversible according to Raman and fluorescence experiments. However, compression above 16 GPa causes the irreversible growth of n IR band in the region of C-H stretching of saturated carbon atoms, which can be interpreted by the formation of permanent intramolecular bonds. On the other hand, H_4_[6]CPP optical properties present a unique response to pressure, with a sharp decrease in the band gap and increase in the lifetime. The lifetimes reach values of 38 ns, with emission in the green region, making these molecules uniquely suitable for use in optoelectronic devices modulated by strain. We believe this behaviour is due to the flexibility that the cyclohexadiene connecting rings provide to the cycle; that is, they can bend to shortening between the intramolecular biphenyl units favouring the π-π interaction while the double bonds of the cyclohexadienes favour conjugation within the molecule as well.

When the phenyl configuration is cyclic, the observed results depend on size. As in the non-conjugated cycle paraphenylene, no irreversibility in the vibrational or optical properties for [12]CPP is seen when compressed up to 12 GPa. It is known that cyclic conjugation decreases with the increasing size of the ring and also that compression induced a change into the circular section towards oval, which occurs at around 1 GPa for [12]CPP [40]. The absorption and fluorescence results presented here demonstrate that compression up to 1 GPa leads to a decrease in the HOMO-LUMO gap of about 0.26 eV. This value is within the calculated difference between a relaxed [12]CPP with alternated torsions and either a cycle with no torsional angles between neighbouring phenyl units or between [12]CPP and [8]CPP with larger cyclic conjugation [49]. Therefore, when [12]CPP is compressed in the rigid circular regime, the cyclic conjugation is favoured. However, at pressures above 1 GPa, compression leads to deformation of the cycle and adoption of an oval configuration.

In the case of [6]CPP, the one with the largest cyclic conjugation and the smallest band gap, its large strain hinders the modulation of these properties. As discussed, pressure induces the formation and asymmetrisation of the cycle, leading to intermolecular interactions that for above 5 GPa have been detected to be irreversible. This work intended to explore the possibility of perturbing the selection rules to activate the S_0_→S_1_ absorption or emission through the deformation of the cycle and perturbation of the selection rules. However, no S_0_→S_1_ absorption or emission was detected either through OP or TP excitations.

This work highlights the fact that different phenyl configurations lead to different physical properties of the materials. Curvature, strain and electronic π-conjugation are the main characteristics of [n]CPPs. [6]LPP exhibits different properties to [6]CPP; i.e., the former shows linear π-conjugation being enhanced by pressure and the latter shows that cyclic conjugation is perturbed by molecular deformations. Although large [n]CPPs are thought to have features close to those of long [n]LPPs, this work demonstrates some important differences between the two systems. The best example is provided by evaluation of the fluorescence of [6]LPP and [12]CPP, as the latter is more affected by compression as it assists the cyclic conjugation that causes an important reduction of its band gap, absorption and emission coefficients more than three times those in[6]LPP.

H_4_[6]CPP was studied with the intention to provide a link between linear and cyclic π-conjugation. Interestingly, here we find that this non-conjugated cyclic system presents attractive optical properties of its own. The closure between biphenyl units and the flexibility of the cyclohexadiene units configures it with exclusive π-conjugation as shown by its characteristic photoluminescence. The H_4_[6]CPP compression leads to intramolecular π-interactions, resulting in an important decrease of the band gap. Therefore, this non-conjugated cycle takes the advantages of the intramolecular deformability of the CPPs and the linear conjugation provided by the biphenyl units, but without the added strain provided by the bending of the phenyl units in the CPPs. Consequently, H_4_[6]CPP configures a great link between linear and cyclic paraphenylenes.

## 4. Materials and Methods

[6]LPP was purchased from TCI. [12]CPP was provided by Yamago et al. and synthesised using a synthetic strategy through multinuclear arylplatinum complexes [29]. [6]CPP and H_4_[6]CPP were provided by Jasti et al. and synthesised through Suzuki–Miyaural cross-coupling/macrocyclisation to generate macrocycles and their consecutive reduction routes [54].

### 4.1. X-ray Crystallography

Single-crystal diffraction data were collected at ambient pressure and room temperature using a crystal of dimensions 0.6 × 0.3 × 0.1 mm^3^ on a Rigaku–Oxford Diffraction SuperNova 4-circle goniometer diffractometer (Wroclaw, Poland) using Cu–Kα radiation (λ = 1.54184 Å).

Diffraction data at high pressure were collected using a crystal of dimensions 0.3 × 0.2 × 0.1 mm^3^, which was loaded together with a ruby chip into a Merrill–Bassett-type diamond anvil cell (DAC) consisting of 600 μm cut diamonds and a tungsten gasket mounted on tungsten–carbide backing seats. The cell was cryo-loaded in argon as a hydrostatic medium. The diffraction experiments were performed at room temperature using silicon(111) monochromated synchrotron radiation (λ = 0.4859 Å) on beamline I19 at the Diamond Light Source, Didcot, UK. Data were collected at 1.43, 1.96, 3.33 and 4.59 GPa and, in all cases, the pressure was measured using the ruby fluorescence method [67].

### 4.2. Structure Analysis

All data were processed and integrated with the program *CrysaAlisPro* [68]. The cell parameters were obtained using Xia2 [69,70,71,72,73].

The multi-scan method was used to treat systematic errors. Structures were solved using direct methods (SHELEXT) [74] and refined by full-matrix least squares of F^2^ (SHELXL) [74] using the Olex2 graphical user interface [75]. Structures were visualised with MERCURY [76].

### 4.3. Interaction Energies Calculation

Molecular electron densities and lattice energies were calculated in CrystalExplorer version 17.5 (University of Western Australia, Perth, Australia) by the embodied TONTO software. The calculations were performed at the B3LYP level with the 6-13G**(d,p) basis set, using molecular geometries from the crystal structures at ambient pressure and 4.59 GPa of H_4_[6]CPP. The C-H bond lengths were normalised to standard neutron diffraction values [12,77].

### 4.4. FTIR

Infrared spectra were measured in the mid-IR region of 600−5000 cm^−1^ with a spectral resolution of 1 cm^−1^ using a Bruker IFS-120HR FTIR spectrometer (Bruker Corporation, Billerica, MA, USA) modified to allow in situ measurements in the DAC [78]. The FTIR spectra were measured just after loading a membrane diamond anvil cell (MDAC) to check the initial sample purity during compression and decompression. Type II diamond anvils were used with diameter culet of 300 μm, using Inconel as gasket material with a final thickness of 40 μm thick with 150 μm hole. Samples were loaded together with a solid solution of KBr and NaNO_2_ (99:1). Pressure was monitored by the fluorescence of a ruby chip placed together with the sample in the chamber.

### 4.5. Raman Spectroscopy

Raman measurements at ambient and approximately hydrostatic high-pressure conditions were conducted with a Senterra dispersive Raman spectrometer from Bruker (Bruker Corporation, Billerica, MA, USA) with a 785 nm excitation wavelength and standard spectral resolution of 3 cm^−1^ [79]. Pressure studies were conducted in a screw-driven sapphire anvil cell (SAC) with anvils of a diameter culet of 380 μm. Pressures up to 12 GPa were reached, although for each [n]CPP, the maximum pressure achieved was slightly different. No PTM was used and diamond chips were employed as a pressure calibrant [80].

### 4.6. Near-UV−Visible Absorption

Near-UV−visible spectra were measured with a homemade spectrometer designed for UV−vis absorption measurements in the anvil cell. The main incorporated components are a CCD - model S9971− 1006UV by Hamamatsu (Photonics K.K., Hamamatsu, Japan), and monochromator - model 77250 by Newport (Newport Corporation, Irvine, CA, USA). The light source was a xenon lamp (Hamamatsu L10725) attenuated by UV-fused silica neutral-density filters with varying optical densities and filtered by a short-pass filter with a cut-off wavelength at 450 nm to reject light out of the desired spectral range. The light was focused onto the sample and collected by a couple of Al-coated 90° off-axis parabolic mirrors (with a reflected focal length of 50.8 mm). Then, the light was focused by Al-coated 90° off-axis parabolic mirrors into a 1/8 m monochromator (Newport 77250), with an F/number = F/3.7. The light was dispersed by a ruled grating (Newport 77304) with 600 lines/mm blazed at 200 nm and collected on a CCD (Hamamatsu S9971−1006UV). The resulting spectral resolution was 3 nm. Pressure-dependent measurements were done using MDAC with ultra-low fluorescence diamonds with diameter culet of 300 μm, using Inconel as gasket, being this of 40 μm thick with 150 μm hole. H4[6]CPP pressure-dependent measurements were done using a screw-driven H-silicon carbide anvil cell, with 400 μm culets, Inconel as gasket being this of 30 μm thick with 100 μm hole. No PTM was used and pressure was monitored by the fluorescence of a ruby chip placed together with the sample in the chamber. Absorption of the anvils with the sample chamber loaded with water was used as reference.

### 4.7. Fluorescence

The fluorescence measurements were performed on polycrystalline samples using a picosecond tunable source and to detect the fluorescence in a backscattering geometry in a setup detailed in Ref. [60]. In the present work, the beam was focused with a 100 mm focal length achromatic doublet to obtain a beam-waist diameter of comparable dimensions with the gasket aperture and a depth of focus longer than the sample thickness. A parabolic Al mirror was used to collect fluorescence, which was focused on a monochromator - model Cornerstone 74100 by Oriel Instruments (now part of Newport corporation, Irvine, CA, USA) and measured by a cooled Hamamatsu R943-02 photomultiplier (Hamamatsu Photonics K.K., Hamamatsu, Japan). Pressure-dependent measurements were done using MDAC provided with ultra-low fluorescence diamonds with diameter culet of 300 μm, using Inconel as gasket, being this of 40 μm thick with 150 μm hole. The sample was loaded with no PTM unless otherwise specified. Pressure was determined by measuring the FTIR spectrum, using the calibrated frequency shift of the infrared absorption bands of the own paraphenylene. Fluorescence spectra were measured with a resolution of ∼1 nm. The sampling of the excitation profiles was set to 1 nm.

## 5. Conclusions

We have demonstrated that phenyl conjugation is a powerful element whose properties can be exploited through the synthesis of attractive structures with different configurations and through mechanical compression. The understanding of this novel and interesting concept provides us with the basic tools needed to synthesise tailored molecular materials with the desired properties.

In this work, we show how different arrangements of phenyl units lead to different pressure modulation of their properties. In [n]LPPs, compression favours linear conjugation through molecular planarisation and intramolecular interactions. In the case of non-conjugated cyclic H_4_[6]CPP, spectroscopic results show how, through compression, conjugation within the molecule is favoured, with the resultant band gap reduction. This may be the most remarkable finding as it opens a broad spectrum of possibilities to use this molecular arrangement in the field of optoelectronic applications. Finally, in the case of strained [n]CPPs, compression of [12]CPP within the low-pressure configuration decreases its band gap; however, once it deforms, the band gap closure narrows and no significant intramolecular rearrangements are observed. In [6]CPP, with higher molecular strain, higher pressures are required to deform the cycle and break the symmetry selection rules; however, there is a tendency of a band gap increase. Such an increase is a consequence of the loss of cyclic conjugation, which is responsible for the low band gap in pristine [6]CPP.

## Figures and Tables

**Table 1:** Interactions in the first coordination sphere of H4{6}CPP at 0 and 4.59 GPa. All energies are in kJ mol^−1^. Color coding correspond to the dummy points marked in Figure 3a.

		Electrostatic		Polarisation		Dispersion		Repulsion		Total	
	Pressure/GPa	0	4.59	0	4.59	0	4.59	0	4.59	0	4.59
***2***	−x, y + 1/2, −z + 1/2	−6.9	−25.1	−1.4	−2.9	−35.9	−66.2	20.4	90.5	−27.1	−30.4
***2***	−x, y + 1/2, −z + 1/2	−6.5	−22.0	−1.4	−2.5	−30.8	−54.1	15.8	70.0	−24.9	−29.0
***2***	x, −y + 1/2, z + 1/2	−0.1	−3.3	−0.1	−0.3	−6.7	−14.3	1.8	17.3	−4.8	−5.6
***2***	x, −y + 1/2, z + 1/2	−8.3	−51.5	−0.6	−1.7	−71.8	−143.5	36.4	197.2	−49.3	−58.9
***2***	x, −y + 1/2, z + 1/2	−4.6	−15.0	−0.8	−2.2	−25.9	−45.5	16.8	74.7	−10.7	−11.0
***1***	−x, −y, −z	−2.5	−29.1	−0.4	−3.1	−13.1	−66.2	3.8	91.1	−12.0	−34.8
***1***	−x, −y, −z	−6.8	−9.5	−1.3	−1.2	−31.5	−33.3	17.2	30.5	−25.0	−21.1
***1***	−x, −y, −z	−3.6	−10.0	−0.5	−1.5	−16.1	−35.4	5.9	34.1	−14.6	−21.5
***1***	−x, −y, −z	−8.9	−33.0	−2.1	−4.1	−47.6	−83.0	29.1	119.9	−34.4	−36.1

**Table 2:** Interceptions and pressure shift of the C-H stretching bands from phenyl contributions.

SYSTEM	ω_0,_ (cm^−1^)	A, (cm^−1^GPa^−1^)
Benzene [44]	2990	4.7 ± 0.2
[6]LPP	3030/3090	5.5 ± 0.4
H_4_[6]CPP	3030	8.0 ± 0.8
[12]CPP	3030/3080	9.0 ± 0.8
[6]CPP	3010/3070	6.8 ± 0.5

**Table 3:** Experimental interceptions and pressure coefficients of the linear variation with pressure of the G bands for each system before (A) and after (B) the ovalisation pressure (*P_oval_*).

			GA_1g_			GE_2g_		
SYSTEM	Diameter (nm)	*P_oval_* (GPa)	ω_0_ (cm^−1^)	A (cm^−1^GPa^−1^)	B (cm^−1^GPa^−1^)	ω_0_ (cm^−1^)	A (cm^−1^GPa^−1^)	B (cm^−1^GPa^−1^)
Benzene [45]	--	--	1567	3.6 ± 0.1	--	--	--	--
[6]LPP	--	--	1592	3.9 ± 0.3	--	--	--	--
H_4_[6]CPP	0.45	9	1610	3.1 ± 0.1	−1.0 ± 0.1	1658	3.2 ± 0.1	--
[12]CPP	1.65 [51]	1	1595	7.0 ± 2.0	3.4 ± 0.1	1602	9.0 ± 2.0	3.9 ± 0.1
[6]CPP	0.81 [52]	5	1567	5.1 ± 0.2	1.0 ± 0.6	1584	4.7 ± 0.2	1.4 ± 0.7

**Table 4:** Experimental interceptions and pressure coefficients of the linear variation with pressure of the absorption bands before (A) and after (B) the ovalisation pressure (*P_oval_).*

		S_0_ → S_1_			S_0_ → S_2_			S_0_ → S_3_-S_4_		
	*P_oval_* (GPa)	λ_0_ (nm)	A (nm/GPa)	B (nm/GPa)	λ_0_ (nm)	A (nm/GPa)	B (nm/GPa)	λ_0_ (nm)	A (nm/GPa)	B (nm/GPa)
Picene [55]	--	475	~8 ± 0.1	--	--	--	--			
[6]LPP	--	325	~1 ± 0.1	--	--	--	--			
H_4_[6]CPP	9	295	~2 ± 0.1	(−1) ± 1			--			
[12]CPP	1	396	~30 ± 0.1	~2 ± 1	344	~15 ± 2	~1 ± 1			
[6]CPP	5	--	--	--	352	~4 ± 1	~(−5) ± 1	326	~8 ± 1	~(−9) ± 1

**Table 5:** Experimental interceptions and pressure coefficients of the linear variation with pressure of the fluorescence bands (The v0-0 has been only considered and, for H_4_[6]CPP, two *PT* are indicated where the different coefficients are: before (A) and after (B) *PT1*, and (C) *after PT_2_*.)

		S_1_ → S_0_			
	*PT* (GPa)	λ_0_ (nm)	A (nm/GPa)	B (nm/GPa)	C (nm/GPa)
Picene [55]		475	~6.0		--
[6]LPP	> 1	440	~2 ± 0.2	--	--
H_4_[6]CPP	4, 9-10	375	~5 ± 1	~20 ± 2	~8 ± 1
[12]CPP	1	462	~50 ± 4	~10 ± 2	--

**Table 6:** Summary of the main results obtained from the different techniques. “—” indicates either there are not data collected or no significant changes are observed through the marked diagnostic.

System	Comments	FTIR	Raman	Absorption	Fluorescence
**[6]LPP**	1 to 2 GPa: planarisation	Spectral changes, new bands [66]	Change in slope of the G_A1g_ mode	Change in the absorption profile	--
	1–23 GPa	Steep upshift C-H stretching modes	--	Steep upshift, measurements conducted up to 12 GPa	Steep upshift measurements conducted up to 12 GPa
	P > 23 GPa: polymerisation	C-H stretching from sp^3^ carbon	--	--	--
**H_4_[6]CPP**	1–4.59 GPa, intramolecular distance decrease, XRD	No significant changes			Change in slope at 5 GPa of the emission bands
	4.6 to 10 GPa: pressure induces π-π interactions	C-H stretching from sp^3^ carbon decrease in intensity	Change in slope of the G_A1g_ mode	Change in slope at 10 GPa of the absorption bands	Change in slope 10 GPa of the emission bands
	P > 16 GPa:polymerisation	C-H stretching from sp^3^ carbon	--	--	--
**[12]CPP**	~0.6 GPa: deformation of the cycle, ovalisation: favoured intramolecular conjugation	--	Change in slope of the G_A1g_ mode	Change in slope of absorption bands; HOMO-LUMO absorption becomes more intense	Change in slope of emission band
	Up to 10 GPa	No significant changes			
**[6]CPP**	5 GPa: Deformation of the cycle and intermolecular polymerisation	C-H stretching from sp^3^ carbon appears in decompression	Change in slope of the G_A1g_ mode and irreversibility on decompression	Change in slope of absorption bands; HOMO-LUMO absorption becomes more intense	None observed

## Supplementary Materials

The following are available online, Figures S1–S10. Table S1.
