# Supplementary material for: Linear, Non-Conjugated Cyclic and Conjugated Cyclic Paraphenylene under Pressure
Source: Molecules. 2019 Sep 26;24(19):3496. doi: 10.3390/molecules24193496 (PMC6803999; doi:10.3390/molecules24193496)
Supplement: Supplementary file 1 [file molecules-24-03496-s001.zip › SoM_8_9_2019.docx]

Supplementary Materials for: Linear, non-conjugated cyclic and cyclic paraphenylene under pressure

Miriam Peña-Álvarez^1^*,Samuele Fanetti^2^, Naomi Falsini^2^, Giulia Novelli^3^, Juan Casado^4,^Valentín G. Baonza^5^, Mercedes Taravillo^5^, Simon Parsons^3^, Roberto Bini^2^, Margherita Citroni^2^

^1^ School of Physics and Astronomy and Centre for Science at Extreme Conditions, University of Edinburgh, Edinburgh EH9 3FD, United Kingdom

^2^ LENS–European Laboratory for Non-Linear Spectroscopy, 50019 Sesto, Florence, Italy;

^3^ Centre for Science at Extreme Conditions and EastChem School of Chemistry and University of Edinburgh, Edinburgh, EH9 3FD, UK

^4^ Department of Physical Chemistry, Faculty of Science, University of Málaga, CEI Andalucía Tech, Campus de Teatinos s/n, 29071 Málaga, Spain

^5^ MALTA-Consolider Team, Department of Physical Chemistry I, Chemistry Faculty, University Complutense of Madrid, 28040 Madrid, Spain

**Table S1**. Crystal packing from single crystal X-ray diffraction for the systems discussed in this work. The crystals of [12]CPP include disordered solvent molecules, their positions have not been refined.

| **[6]LPP,** Heimel et al. ^[[1]](#endnote-1)^ |  |
| --- | --- |
| 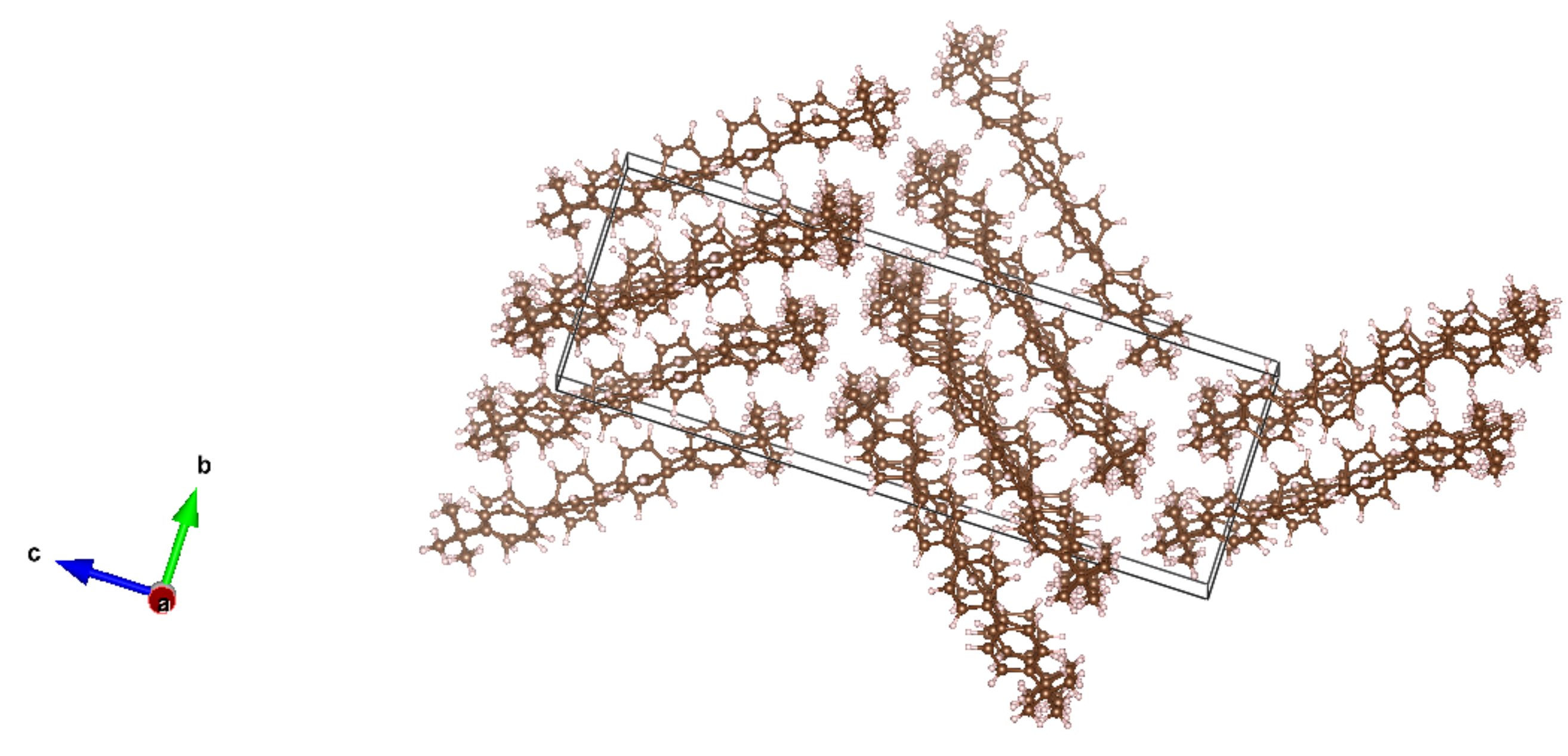 | 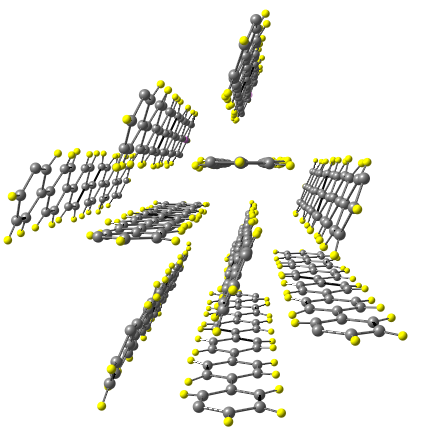 |
| 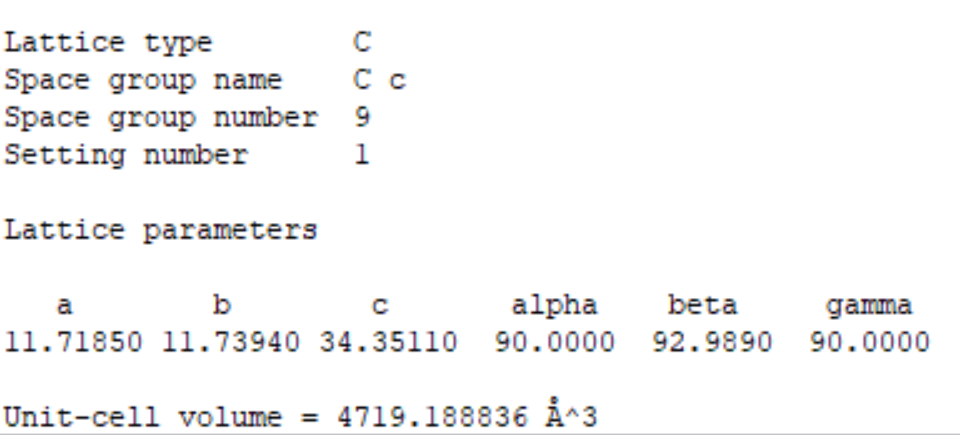 |   Figure and data taken from Heimel et al.[1] View along the long axes of molecules within one layer of a *para*-terphenyl crystal. The herringbone angle *θ* is defined as the angle between the planes of two translationally inequivalent molecules. Upon applying hydrostatic pressure, the unit cell axis *a* shortens twice as much as the unit cell axis *b* and the molecules twist around their long axis as indicated by the curved arrows. |

**Table S2**. Crystal packing from single crystal X-ray diffraction [12]- and [6]CPP. The crystals of [12]CPP include disordered solvent molecules, their positions have not been refined.

| **[12]CPP**  **Ref.** **[****^[[2]](#endnote-2)^]** | Space group | Monoclinic  P 21/c | 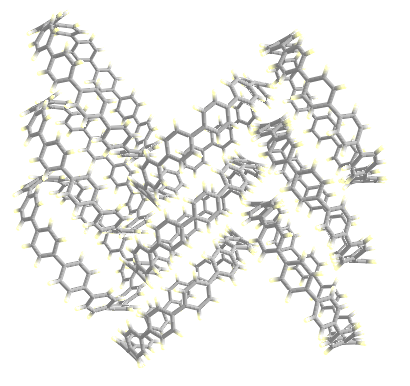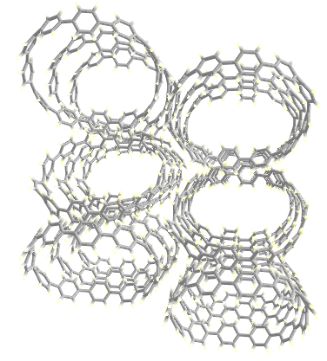 |
| --- | --- | --- | --- |
|  | a | 18.5827(7) Å |  |
|  | b | 8.1878(3) Å |  |
|  | c | 23.6701(9) Å |  |
| **[6]CPP**  **Ref. [^[[3]](#endnote-3)^]** | Space group | Hexagonal, R-3 | 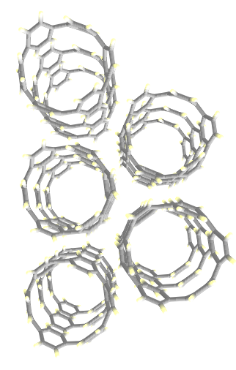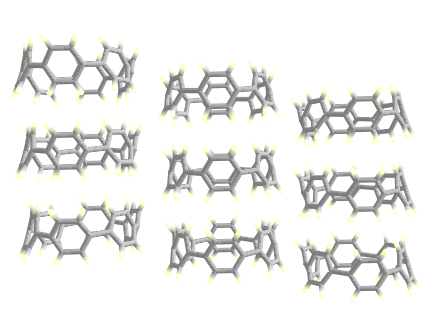 |
|  | **a** | 19.3957 (4) Å |  |
|  | **c** | 6.1998 (2) Å |  |

**Table S3. Experimental details** for single crystal X-ray diffraction for H_4_[6]CPP measured in this work. For all structures: C_36_H_28_, *M*_r_ = 460.61, monoclinic, *P*2_1_/*c*, *Z* = 4. Absorption was corrected for by multi-scan methods, *CrysAlis PRO* 1.171.39.46 (Rigaku Oxford Diffraction, 2018) Empirical absorption correction using spherical harmonics, implemented in SCALE3 ABSPACK scaling algorithm. H-atom parameters were constrained.

| Code in ESI | mgra00 | mgra01 | mgra02 | mgra03 | mgra04 |
| --- | --- | --- | --- | --- | --- |
| CCDC number | 1950352 | 1950353 | 1950354 | 1950355 | 1950356 |
| **Crystal data** | | | | |  |
| Temperature (K) | 295 | 293 | 293 | 293 | 293 |
| Pressure (GPa) | 0 | 1.43 | 1.96 | 3.33 | 4.60 |
| *a*, *b*, *c* (Å) | 14.3610 (9), 13.8896 (7), 12.5892 (7) | 14.1470 (6), 13.0300 (7), 12.033 (2) | 14.0923 (5), 12.8090 (6), 11.909 (2) | 13.9882 (6), 12.4476 (6), 11.710 (2) | 13.9268 (17), 12.245 (2), 11.591 (6) |
| β (°) | 102.609 (6) | 101.838 (7) | 101.769 (7) | 101.698 (8) | 101.81 (2) |
| *V* (Å^3^) | 2450.6 (2) | 2171.0 (4) | 2104.5 (4) | 1996.6 (4) | 1934.8 (11) |
| Radiation type | Cu *K*α | Synchrotron,  = 0.4859 Å | Synchrotron,  = 0.4859 Å | Synchrotron,  = 0.4859 Å | Synchrotron,  = 0.4859 Å |
|  (mm^-1^) | 0.53 | 0.04 | 0.04 | 0.05 | 0.05 |
| Crystal size (mm) | 0.4 × 0.2 × 0.1 | 0.3 × 0.2 × 0.1 | 0.3 × 0.2 × 0.1 | 0.3 × 0.2 × 0.1 | 0.3 × 0.2 × 0.1 |
|  | | | | |  |
| **Data collection** | | | | |  |
| Diffractometer | SuperNova, Dual, Cu at home/near, Atlas | Beamline I19 EH2 at Diamond Light Source | Beamline I19 EH2 at Diamond Light Source | Beamline I19 EH2 at Diamond Light Source | Beamline I19 EH2 at Diamond Light Source |
| *T*_min_, *T*_max_ | 0.674, 1.000 | 0.884, 1.000 | 0.252, 1.000 | 0.751, 1.000 | 0.576, 1.000 |
| No. of measured, independent and observed [*I* > 2σ(*I*)] reflections | 20243, 5049, 3735 | 9800, 1969, 1458 | 12570, 1843, 1284 | 10872, 1582, 1034 | 4933, 637, 317 |
| *R*_int_ | 0.057 | 0.059 | 0.059 | 0.063 | 0.104 |
| _max_ (°) | 76.1 | 15.8 | 15.3 | 14.9 | 11.3 |
| (sin /λ)_max_ (Å^-1^) | 0.630 | 0.559 | 0.542 | 0.530 | 0.403 |
| Completeness | 0.999 | 0.618 | 0.657 | 0.634 | 0.602 |
|  | | | | |  |
| **Refinement** | | | | |  |
| *R*[*F*^2^ > 2σ(*F*^2^)], *wR*(*F*^2^), *S* | 0.063, 0.187, 1.02 | 0.048, 0.122, 1.07 | 0.063, 0.177, 1.09 | 0.068, 0.167, 1.14 | 0.087, 0.310, 1.07 |
| No. of reflections | 5049 | 1969 | 1843 | 1582 | 637 |
| No. of parameters | 326 | 325 | 325 | 325 | 145 |
| No. of restraints | 688 | 688 | 688 | 688 | 382 |
|  | *w* = 1/[σ^2^(*F*_o_^2^) + (0.1075*P*)^2^ + 0.1626*P*]  where *P* = (*F*_o_^2^ + 2*F*_c_^2^)/3 | *w* = 1/[σ^2^(*F*_o_^2^) + (0.0445*P*)^2^ + 1.1936*P*]  where *P* = (*F*_o_^2^ + 2*F*_c_^2^)/3 | *w* = 1/[σ^2^(*F*_o_^2^) + (0.0311*P*)^2^ + 8.3171*P*]  where *P* = (*F*_o_^2^ + 2*F*_c_^2^)/3 | *w* = 1/[σ^2^(*F*_o_^2^) + (0.0217*P*)^2^ + 10.1855*P*]  where *P* = (*F*_o_^2^ + 2*F*_c_^2^)/3 | *w* = 1/[σ^2^(*F*_o_^2^) + (0.1128*P*)^2^ + 32.0008*P*]  where *P* = (*F*_o_^2^ + 2*F*_c_^2^)/3 |
| ρ_max_, ρ_min_ (Å^-3^) | 0.25, -0.22 | 0.13, -0.13 | 0.24, -0.33 | 0.22, -0.23 | 0.23, -0.29 |

**Table S4**. Crystal packing from single crystal X-ray diffraction for H_4_[6]CPP measured in this work.

| **H_4_[6]CPP**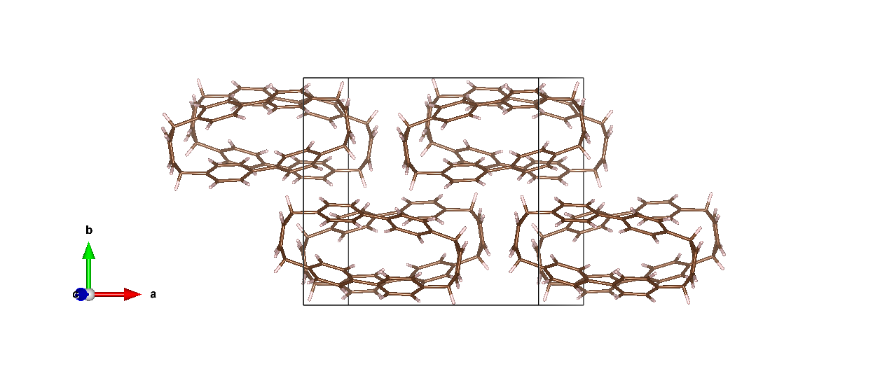 | | 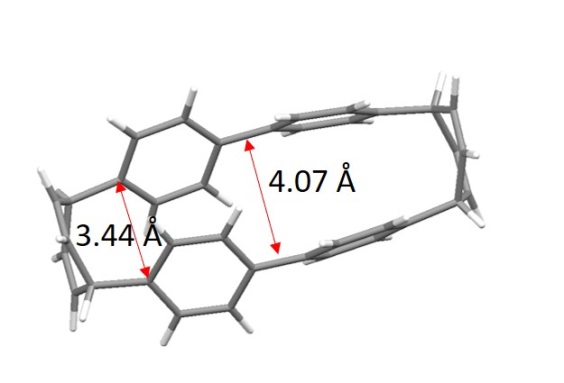 |
| --- | --- | --- |
| Space group | Orthorombic P2_1_/c |  |
| Z:4 | Z’:0 |  |
| R factor (%) | 6.11 |  |
| a_0_ | 14.361 (9) Å |  |
| b_0_ | 13.8896(7) Å |  |
| c_0_ | 12.5892(7) Å |  |
| β_0_ | 102.609(6)° |  |
| V_0_ | 2450.6(3) Å^3^ |  |
| **The effect of pressure on the topology of packing in H_4_{6}CPP**  At ambient pressure, the molecular coordination number of H_4_[6]CPP is 14. As suggested by the Voronoi-Dirichlet analysis, the topology resembles a distorted version of the body-centred cubic packing and shows a coordination sequence of 14-50-110. At 4.59 GPa, while the first coordination sphere stays the same, the distortion is replaced by a more regular geometry and the coordination sequence becomes 14-52-116. A comparison between the archetypal BCC topology of tungsten with H_4_[6]CPP structures at 0 and 4.59 GPa is represented in *the next figure*  All the structure representations are viewed along the c-axis. The H_4_[6]CPP topology (i), which deeply reminds the prefect BCC packing of Tungsten, becomes more geometric and regular upon compression. Voronoi-Dirichlet polyhedral represent packing topology in Tungsten (ii), in H_4_[6]CPP at ambient pressure (iii) and 4.59 GPa (iv). 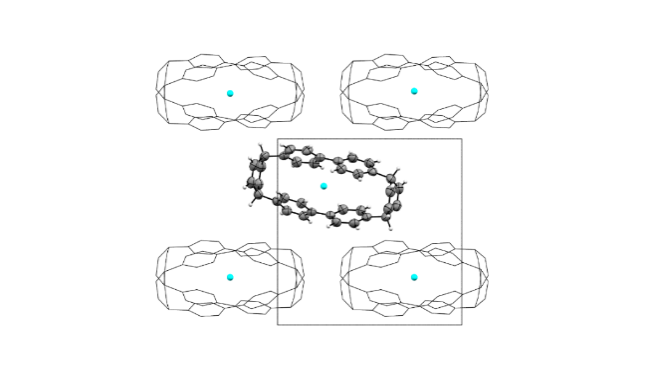 *c*  *a*  *b* 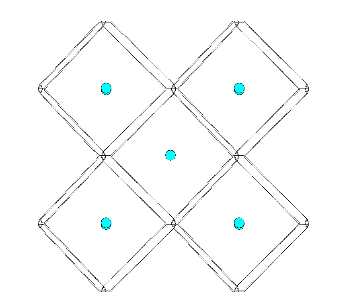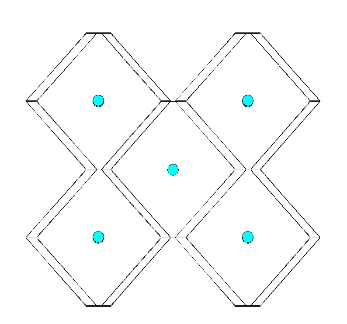 ***iii iv*** 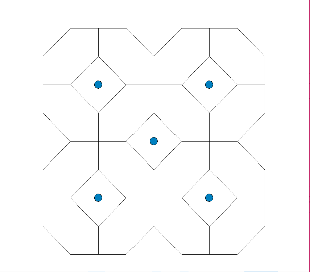 ***i ii*** | | |

Figure S1. (a) Calculated allowed vertical transition for the geometry optimized structure of twisted [6]LPP and for the structure with no torsional angle between neighboring phenyl units. Optimized structures are taken from ref. [^[[4]](#endnote-4)^]. (b-d) FTIR absorption spectra of [6]LPP at different pressures. Arrows are used to indicate spectral changes.

[6]LPP. at room conditions belongs to the D_2_ symmetry group. FTIR high pressure experiments [6]LPP which confirm that a transition towards a different molecular symmetry occurs at pressures below 1 GPa. Between 7 and 12 GPa a growth and intensity decrease of certain bands in the CH wagging region (800 cm^-1^) is observed and related with a phase transition toward D_2h_ configuration as this band is characteristic of changes in the conjugation within the chain. [^[[5]](#endnote-5)^]

Figure S2. FTIR absorption spectra of H_4_[6]CPP at different pressures. From bottom to top pressure is increased up to 21.3 GPa and then decreased to 0.5 GPa (red spectra).

Figure S3. FTIR absorption spectra of [12]CPP at different pressures. From bottom to top pressure is increased up to 10.7 GPa and then decreased to 0.1 GPa (red spectra).

Figure S4. FTIR absorption spectra of [6]CPP at different pressures. From bottom to top pressure is increased up to 6.7 GPa and then decreased to 0.7 GPa (red spectra).

Figure S5. (a) Raman spectra in the 1600 cm^-1^ region at different pressures for [6]LPP. (b) Raman shift as a function of pressure. Different symbols correspond to different experimental runs. Line correspond to the linear fit of the experimental points vs pressure.

Figure S6. Raman spectra in the 1600 cm^-1^ region at different pressures for H_4_[6]CPP. Right, Raman shift as a function of pressure. Different symbols correspond to different experimental runs. Lines correspond to fitting to eq 1 of the main text of the experimental points vs pressure. Red contribution corresponds to the C-C stretching mode of the phenyl units, while green ones to the C=C stretching from the cyclohexadiene units.

Figure S7. Raman spectra in the 1600 cm^-1^ region at different pressures for [12]CPP. Right, Raman shift as a function of pressure. Different symbols correspond to different experimental runs. Lines correspond to fitting to eq 1 of the main text of the experimental points vs pressure. Red contribution corresponds to the C-C stretching mode along the transversal direction, while the blue is along the longitudinal one mainly.

Figure S8. Raman spectra in the 1600 cm^-1^ region at different pressures for [6]CPP. Right, Raman shift as a function of pressure. Different symbols correspond to different experimental runs. Lines correspond to fitting to eq 1 of the main text of the experimental points vs pressure. Red contribution corresponds to the C-C stretching mode along the transversal direction, while the blue is along the longitudinal one mainly

Since the FTIR results showed that compression of H_4_[6]CPP up to 17 GPa results in the formation of a product with C of higher sp^3^ character, the turnover in the B slope of the G mode could be interpreted as the point at which the π-π distances become close enough to lead towards intramolecular σ formation. However, as seen in figure S9, the decompression product in the experimental run at 10 GPa shows full reversibility, demonstrating that the pressure threshold for σ bond formation in this H_4_[6]CPP is between 10 and 17 GPa.


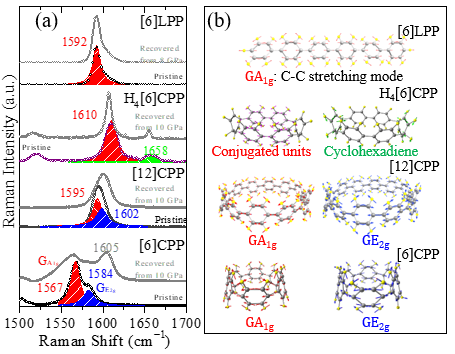


Figure S9. (a) Low pressure Raman spectra in the 1600 cm^-1^ region of the different systems here studied before and after compression (b) Molecular schemes with the eigenvectors involved in each of ther marked modes.

Figure S10. Calculated allowed vertical transition for the geometry optimized structure of twisted [6]LPP and for the structure with no torsional angle between neighboring phenyl units.

***Excitation energies and oscillator strengths for H4[6}CPP, DFT TD-BrLYP/6-31(d,p_:***

Excited State 1: Singlet-AG 4.4673 eV 277.54 nm f=0.0000 <S**2>=0.000

121 ->124 0.12157

122 ->123 0.68945

This state for optimization and/or second-order correction.

Total Energy, E(TD-HF/TD-KS) = -1388.49380052

Copying the excited state density for this state as the 1-particle RhoCI density.

Excited State 2: Singlet-AU 4.5783 eV 270.81 nm f=0.0160 <S**2>=0.000

121 ->123 0.56060

122 ->124 -0.42899

Excited State 3: Singlet-AG 4.6268 eV 267.97 nm f=0.0000 <S**2>=0.000

121 118 ->124 0.15766

120 ->123 -0.32654

121 ->126 -0.27289

122 ->125 0.50866

Excited State 4: Singlet-AU 4.7877 eV 258.96 nm f=0.0016 <S**2>=0.000

118 ->124 0.15766

120 ->123 -0.32654

121 ->126 -0.27289

122 ->125 0.50866

Excited State 5: Singlet-AU 4.8024 eV 258.17 nm f=0.9114 <S**2>=0.000

121 ->123 0.40337

122 ->124 0.53508

122 ->128 0.11041

Excited State 8: Singlet-AU 4.9485 eV 250.55 nm f=0.0577 <S**2>=0.000

112 ->123 -0.10236

117 ->123 -0.26084

119 ->124 0.32750

121 ->127 0.42786

122 ->124 0.12639

122 ->128 -0.29326

Excited State 10: Singlet-AU 5.1543 eV 240.55 nm f=0.0177 <S**2>=0.000

120 ->123 0.13485

121 ->126 0.55695

122 ->125 0.40548

Excited State 11: Singlet-AU 5.2967 eV 234.08 nm f=0.0317 <S**2>=0.000

121 ->127 0.45605

122 ->128 0.52489

Excited State 16: Singlet-AU 5.3563 eV 231.47 nm f=0.0126 <S**2>=0.000

116 ->123 -0.10145

122 ->130 0.68514

Excited State 20: Singlet-AU 5.4705 eV 226.64 nm f=0.0008 <S**2>=0.000

116 ->123 0.43892

117 ->123 -0.42286

119 ->124 -0.33738

Excited State 22: Singlet-AU 5.4775 eV 226.35 nm f=0.0303 <S**2>=0.000

116 ->123 0.53238

117 ->123 0.33771

119 ->124 0.29478

Excited State 23: Singlet-AU 5.4966 eV 225.57 nm f=0.0443 <S**2>=0.000

115 ->124 0.23467

118 ->124 0.56544

120 ->123 -0.16658

121 ->126 0.16217

121 ->129 0.15720

122 ->125 -0.13626

Excited State 26: Singlet-AU 5.6302 eV 220.21 nm f=0.0020 <S**2>=0.000

114 ->123 0.14057

115 ->124 0.61783

118 ->124 -0.14872

121 ->126 -0.10954

121 ->129 -0.10531

Excited State 27: Singlet-AU 5.6414 eV 219.77 nm f=0.0602 <S**2>=0.000

111 ->125 -0.12621

112 ->123 0.25721

114 ->126 0.10732

117 ->123 -0.30335

119 ->124 0.36873

121 ->127 -0.24697

122 ->128 0.27762

Excited State 29: Singlet-AU 5.6891 eV 217.93 nm f=0.0022 <S**2>=0.000

114 ->123 0.57042

115 ->124 -0.10842

121 ->131 -0.27224

122 ->132 -0.19587

Excited State 32: Singlet-AU 5.7996 eV 213.78 nm f=0.0156 <S**2>=0.000

112 ->123 0.55939

114 ->126 -0.10022

118 ->125 0.30643

121 ->127 0.13353

122 ->128 -0.11435

Excited State 33: Singlet-AU 5.8176 eV 213.12 nm f=0.0050 <S**2>=0.000

111 ->124 -0.11311

113 ->124 0.40541

119 ->125 0.21094

121 ->131 -0.11890

122 ->132 0.44697

Excited State 37: Singlet-AU 5.8806 eV 210.84 nm f=0.0504 <S**2>=0.000

112 ->123 -0.25458

115 ->125 0.16297

116 ->127 -0.25734

118 ->125 0.51350

119 ->128 0.11839

122 ->128 0.10022

122 ->133 0.15693

Excited State 40: Singlet-AU 5.9212 eV 209.39 nm f=0.0074 <S**2>=0.000

111 ->124 0.10666

113 ->124 0.14812

114 ->123 -0.17976

118 ->128 0.11884

119 ->125 0.24121

120 ->127 0.48651

121 ->131 -0.26800

122 ->132 -0.19180

Excited State 41: Singlet-AU 5.9276 eV 209.16 nm f=0.0261 <S**2>=0.000

115 ->125 0.11463

117 ->127 0.23177

119 ->130 0.18577

120 ->126 0.60405

120 ->129 -0.12567

Excited State 47: Singlet-AU 5.9999 eV 206.64 nm f=0.0110 <S**2>=0.000

111 ->124 -0.22392

114 ->123 0.18143

115 ->128 0.14356

117 ->126 0.42052

118 ->128 -0.10850

118 ->130 0.14510

119 ->125 -0.17023

120 ->127 0.30329

121 ->131 0.16522

Excited State 49: Singlet-AU 6.0133 eV 206.18 nm f=0.0335 <S**2>=0.000

115 ->125 0.51567

116 ->127 -0.25854

118 ->125 -0.18986

119 ->128 -0.27259

120 ->129 0.10514

122 ->133 -0.11639

Excited State 50: Singlet-AU 6.0280 eV 205.68 nm f=0.0086 <S**2>=0.000

114 ->126 -0.17493

119 ->128 -0.12286

122 ->133 0.62890

Excited State 51: Singlet-AU 6.0399 eV 205.28 nm f=0.0229 <S**2>=0.000

111 ->124 -0.14691

115 ->128 0.10326

115 ->130 0.10860

116 ->126 -0.14809

116 ->129 -0.32124

117 ->129 -0.15666

118 ->128 0.30082

118 ->130 0.34864

120 ->127 -0.13535

121 ->131 -0.15632

Excited State 54: Singlet-AU 6.0608 eV 204.57 nm f=0.0073 <S**2>=0.000

111 ->124 -0.32477

113 ->124 0.17882

113 ->128 -0.10100

114 ->123 0.10957

115 ->128 -0.23850

115 ->130 0.10791

116 ->126 0.39820

117 ->126 -0.22222

120 ->127 0.12358

Excited State 56: Singlet-AU 6.0758 eV 204.06 nm f=0.0054 <S**2>=0.000

117 ->127 0.25485

119 ->128 0.10348

119 ->130 -0.36537

120 ->126 0.12970

120 ->129 0.49900

Excited State 58: Singlet-AU 6.1374 eV 202.02 nm f=0.0334 <S**2>=0.000

111 ->124 0.34558

116 ->126 0.30360

117 ->126 0.22428

117 ->129 -0.16719

118 ->128 -0.19096

118 ->130 0.25573

120 ->127 -0.11853

121 ->131 -0.14703

122 ->132 0.14030

1. Heimel, G., Puschnig, P., Oehzelt, M., Hummer, K., Koppelhuber-Bitschnau, B., Porsch, F., Ambrosch-Draxl, C. Resel, R.,. *J. Phys.: Cond. Mat.* **2003,** *15*, .3375. [↑](#endnote-ref-1)
2. ### . Segawa, Y.; Miyamoto, S.; Omachi, H.; Matsuura, S.; Senel, P.; Sasamori, T.; Tokitoh, N.; Itami, K. Concise synthesis and crystal structure of [12] cycloparaphenylene. *Angew. Chem., Int. Ed.* **2011**, *50* (14)*,* 3244–3248.

   [↑](#endnote-ref-2)
3. ### . Xia, J.; Jasti, R. Synthesis, characterization, and crystal structure of [6] cycloparaphenylene *Angew*. *Chem., Int. Ed.* **2012**, *51* (10), 2474 –2476.

   [↑](#endnote-ref-3)
4. Peña-Alvarez, M.; Qiu, L.; Taravillo, M.; Baonza, V. G.; Ruiz Delgado, M. C.; Yamago, S.; Jasti, R. López Navarrete, J. T.; Casado, J.; Kertesz, M. *Phys. Chem. Chem. Phys.* **2016***, 18*, 11683-11692. [↑](#endnote-ref-4)
5. G. Froyer, J. Y. Goblot, J. L. Guilbert, F. Maurice, Y. Pelows, *J. Phys.. Colloq.* **1983**, *44*, C3-745 [↑](#endnote-ref-5)
